# Use of oxidized regenerated cellulose to achieve hemostasis during laparoscopic cholecystectomy: a retrospective cohort analysis

**DOI:** 10.1186/s13104-018-3344-3

**Published:** 2018-04-11

**Authors:** Emilia Masci, Giuseppe Faillace, Mauro Longoni

**Affiliations:** Division of General Surgery, Ospedale Edoardo Bassini, ASST Nord Milano, Via Gorki 50, 20092 Cinisello Balsamo, MI Italy

**Keywords:** Bleeding, Laparoscopic cholecystectomy, Hemorrhage, Hemostasis, Oxidized regenerated cellulose, Topical hemostat

## Abstract

**Objective:**

Laparoscopic cholecystectomy is the first-choice treatment for symptomatic cholelithiasis. Though generally safe, this procedure is not without complications, with bleeding the most frequent cause of conversion to open cholecystectomy. Oxidized regenerated cellulose (ORC) added to conventional hemostatic strategies, is widely used to control bleeding during surgery despite limited evidence supporting its use. This retrospective study analyzed patients undergoing laparoscopic cholecystectomy in an Italian center over a 16-month period, between October 2014 and February 2016, who experienced uncontrollable bleeding despite the use of conventional hemostatic strategies, requiring the addition of ORC gauze (Emosist^®^).

**Results:**

Of the 530 patients who underwent laparoscopic cholecystectomy, 24 (4.5%) had uncontrollable bleeding from the liver bed. Of these, 62.5% had acute cholecystitis and 33.3% chronic cholecystitis; 1 patient was diagnosed with gallbladder carcinoma, postoperatively. Most patients had comorbidities, 16.7% had liver cirrhosis, and 37.5% used oral anticoagulants. The application of ORC rapidly controlled bleeding in all patients. Patients were discharged after a mean duration of 2.2 days. ORC was easy to use and well tolerated. Bleeding complications remain a relevant issue in laparoscopic cholecystectomy. ORC was able to promptly stop bleeding not adequately controlled by conventional methods and appears, therefore, to be a useful hemostat.

## Introduction

Laparoscopic cholecystectomy has been the first-choice treatment for symptomatic cholelithiasis, and is among the most frequently performed interventions [1, 2]. Compared with open surgery, laparoscopic cholecystectomy has important advantages, including reduced morbidity and mortality, shorter hospital stay, faster return to daily activities, better cosmetic results, and reduced postoperative pain [3–5]. Laparoscopic cholecystectomy can also be offered to patients with acute cholecystitis [6–8] or cirrhosis, two conditions traditionally considered as contraindications for laparoscopic surgery due to an increased risk of complications [9–11].

Despite its generally favorable safety profile, laparoscopic cholecystectomy is not without complications [12], and rates of up to 10% for conversion to open cholecystectomy are still reported [13]. Bile duct injuries are the most feared complication due to substantial morbidity [14–16]. Bleeding complications, during surgery or postoperatively, are also relevant in laparoscopic cholecystectomy, but less attention has been devoted to them [14]. The lack of a generally accepted classification of bleeding events may have contributed to this situation.

Various tools and procedures are currently available to ensure hemostasis during surgical interventions [17]. Conventional strategies include electrocautery and suture ligation [17], however, the prolonged use of bipolar or monopolar electrocautery in laparoscopic cholecystectomy has been associated with damage to parenchymal tissue and postoperative bile leakage, and should be avoided [18]. Several topical hemostats including oxidized regenerated cellulose (ORC), gelatin, collagen, thrombin, fibrin sealants, and synthetic sealants are also available for a variety of clinical settings [17, 19].

ORC, developed over 60 years ago, is a plant-derived, passive hemostat that promotes hemostasis by providing a matrix for platelet adhesion and aggregation. It is available in various fabric formats allowing for multiple applications, can conform to irregular surfaces and hard-to-reach sites, adheres easily to bleeding surfaces, and is suitable for laparoscopic cholecystectomy [20]. ORC is rapidly absorbed by hydrolysis within 7–20 days depending on the amount used. [21, 22]. ORC has bactericidal properties attributable to its local pH-lowering effect against a wide spectrum of pathogens, including methicillin-resistant *Staphylococcus aureus* [23, 24]. ORC has been evaluated in prospective randomized studies only recently, despite its widespread use in surgical practice [25–27], hence evidence-based information on its optimal use is lacking.

This retrospective analysis reports on our experience with ORC for hemostasis in patients undergoing laparoscopic cholecystectomy who experienced uncontrollable bleeding despite the use of conventional hemostatic strategies.

## Main text

### Methods

#### Study design and patients

This was a retrospective analysis of data from patients who underwent laparoscopic cholecystectomy at the Division of General Surgery of the Edoardo Bassini Hospital, Cinisello Balsamo, Italy, between October 2014 and February 2016. The primary objective was to describe the use and outcomes of topical hemostasis with ORC in patients with difficult-to-treat bleeding during laparoscopic cholecystectomy. Difficult-to-treat bleeding was assessed based on the bleeding flow and depth during laparoscopic cholecystectomy. If bleeding continued uncontrolled for a few minutes despite the use of conventional hemostatic techniques (monopolar or bipolar electrocautery), ORC gauze was applied on the liver bed to avoid prolonged use of electrocautery in order to prevent parenchymal tissue damage. Conventional hemostatic techniques were standardized and consistent across all patients. Clip or suture blind application during major bleeding should be avoided due to high risk of associated vascular and biliary damage.

Data were extracted from the surgical records of the center, including information related to hemostatic procedures performed during the intervention. Patients who experienced bleeding from the liver bed that could not be adequately controlled by conventional hemostatic techniques, and required the addition of a topical hemostatic agent, were included. The hemostatic agent used was ORC gauze (Emosist^®^, Mascia Brunelli, Milano, Italy). Patients, who required conversion to open surgery because of major bleeding (i.e., involving the major branch of the middle hepatic vein), or bile duct injury, were not included in the analysis. The following data were extracted: demographic characteristics; type of gallbladder disease; comorbidities; risk factors for bleeding (cirrhosis, use of anticoagulants); need for transfusions; need for a new intervention; duration of hospital stay; hospital readmission. The analysis was performed after study approval by the local Institutional Review Board P.O. Edoardo Bassini, ASST Nord Milano. All patients provided their written informed consent for being included in this study.

#### Interventions

Diagnostic procedures and cholecystectomy were performed according to current national and international guidelines for cholelithiasis management and laparoscopic cholecystectomy [1, 2, 9, 28]. Diagnosis of acute or chronic cholecystitis was based on medical history, physical examination, laboratory tests, and ultrasound imaging. In both early and elective laparoscopic cholecystectomy, the pneumoperitoneum was established by inserting a Veress needle in the umbilical region followed by the introduction of four trocars. Adhesions, when present, were severed to ensure the optimal exposure of the surgery field and identification of the cystic artery and cystic duct in the Calot’s triangle. Cystic artery and cystic duct were ligated with clips before division, and dissection of the gallbladder from the liver bed was performed using a monopolar electrocautery hook.

During the procedure, hemostasis was ensured by electrocautery (bipolar clamp), if needed. In patients with persistent bleeding despite bipolar electrocautery, a 10 × 20 cm folded piece of ORC gauze was placed in the liver bed. After achievement of bleeding control, the liver bed was monitored for at least 5 min and blood clots were removed using an irrigation-aspiration system. Although we routinely adopt a no-drain policy for laparoscopic cholecystectomy, patients with persistent bleeding during surgery received a sub-hepatic drainage. After the intervention, patients were kept under observation for at least 48 h before hospital discharge.

Blood samples were taken preoperatively and prothrombin time (PT), activated partial thromboplastin time, international normalized ratio (INR) and platelets were measured in all patients undergoing laparoscopic cholecystectomy. Patients receiving anticoagulants at presentation and undergoing elective laparoscopic cholecystectomy, discontinued oral anticoagulants before surgery and switched to low molecular weight heparin. Patients on oral anticoagulants needing early laparoscopic cholecystectomy due to acute cholecystitis received transfusions of fresh frozen plasma, intravenous vitamin K and/or prothrombin complex concentrate, according to clinical condition. No blood samples were taken intra-operatively as it was presumed that all hemostatic parameters were within normal limits in all patients.

#### Statistical methods

Data were analyzed by descriptive statistics and summarized as percentages or mean values.

### Results

In the 16-month period, 530 patients underwent laparoscopic cholecystectomy in our center. The use of ORC gauze during surgery was required in 24 (4.5%) patients [10 males and 14 females, median age 58 years (range 18–84)], as conventional methods failed to adequately control bleeding from the liver bed. In all 24 cases, effective hemostasis was typically achieved between 3 and 7 min from application of the ORC gauze. Patients’ demographic and preoperative characteristics are shown in Table 1. The majority of patients (15/24, 62.5%) underwent early laparoscopic cholecystectomy due to acute cholecystitis, whereas the intervention was elective for 8 patients (33.3%) with chronic cholecystitis; 1 patient who underwent laparoscopic cholecystectomy for suspected chronic cholecystitis was postoperatively diagnosed with advanced gallbladder cancer (pT2). Among patients with chronic cholecystitis, 3 (37.5%) had a history of endoscopic retrograde cholangiopancreatography plus sphincterotomy for biliary pancreatitis. In 2 of the 8 patients with chronic cholecystitis, the procedure was complicated by the presence of scarred tissue. In these patients, the gallbladder was intentionally ruptured and partially removed; the portion that could not be dissected was left in the liver bed. Nonetheless, the application of ORC to the liver bed after dissection provided adequate hemostasis with no need for hemostatic suture in the liver bed and, more importantly, for conversion to open cholecystectomy. Most patients (20/24, 83.3%) had comorbidities including hypertension, heart disease, diabetes, chronic renal insufficiency, and chronic liver disease. Four patients (16.7%) had liver cirrhosis, which was hepatitis B virus-related in 3 patients and hepatitis C virus-related in 1 patient. In nine patients (37.5%) receiving oral anticoagulants, laparoscopic cholecystectomy was performed in the presence of normal values of PT and INR.

**Table 1:** Patient demographic and clinical characteristics (N = 24)

Age, years (range)	58 (18–84)
Female, n (%)	14 (58.3)
Male, n (%)	10 (41.7)
Gallbladder disease, n (%)	
Acute cholecystitis	15 (62.5)
Chronic cholecystitis	8 (33.3)
Gallbladder carcinoma	1 (4.2)
With comorbidities, n (%)	20 (83.3)
With liver cirrhosis, n (%)	4 (16.7)
In treatment with oral anticoagulants, n (%)	9 (37.5)

Two patients (8.3%) who had undergone emergency laparoscopic cholecystectomy due to acute cholecystitis, required blood transfusions for 48 h postoperatively. The mean duration of hospital-stay after laparoscopic cholecystectomy was 2.2 days for the overall population. There were no reoperations and no readmissions after discharge. Diagnostic procedures performed postoperatively and at hospital discharge did not detect any abscess or granuloma.

### Discussion

Intraoperative bleeding remains a relevant complication in laparoscopic cholecystectomy. In line with reported incidence rates [14, 29], 4.5% of patients in this retrospective study experienced a bleeding event that could not be controlled by conventional hemostatic strategies. Our data show that the use of ORC provided adequate hemostasis during the intervention preventing conversion to open surgery, or prolonged and potentially damaging use of conventional hemostatic strategies, such as electrocautery. In our experience, the laparoscopic application of ORC gauze to the liver bed was easy to perform and resulted in effective bleeding control within minutes.

Recently, fewtrials and observational reports have tried to define the efficacy and safety of ORC and other topical hemostatic agents in a variety of surgery settings, including hepatectomy [30, 31], neurosurgery [25], gynecologic surgery [32, 33], nephrectomy [34, 35], and thyroid surgery [36, 37]. To our knowledge, this is the first study to investigate the efficacy and safety of ORC gauze in laparoscopic cholecystectomy.

The application of ORC gauze in our patients was well tolerated, without adverse events. However, ORC is limited by its potential to swell causing pressure on adjacent tissues and organs, hence it should be removed after surgery when used in proximity of sensitive areas, especially in neurosurgery [25]. Rarely, foreign-body reactions to ORC may occur, which can be erroneously interpreted as postoperative abscesses on computed tomography (CT) [32, 33, 38–42]. Therefore, the use and location of ORC should be reported in the patient chart and notified to other treating clinicians, to avoid misinterpretation of CT findings and unnecessary diagnostic interventions [32, 38].

Perhaps not surprisingly, most patients with uncontrolled bleeding in our study were those undergoing early laparoscopic cholecystectomy due to acute cholecystitis. Previously, acute cholecystitis was considered a contraindication for laparoscopic intervention, as the inflammatory environment leads to neoangiogenesis in the liver bed and to increased risk of bleeding. In all our patients with acute cholecystitis and uncontrolled bleeding with traditional hemostatic methods, application of ORC gauze to the bleeding site successfully stopped bleeding.

One-third of our patients with uncontrolled bleeding had chronic cholecystitis. In chronic forms of gallbladder disease, especially if previously treated with conservative therapies including antibiotics and reduced food intake, fibrotic and scarred tissue in the liver bed complicates gallbladder dissection and increases the risk of bleeding. Our results show ORC gauze was effective in ensuring adequate bleeding control in these chronic cases, including two cases involving intentional gallbladder rupture where the portion that could not be removed without high risk of vascular and biliary injury was left in the liver bed.

In uncomplicated laparoscopic cholecystectomy, the postoperative use of an abdominal drain is generally not recommended, lacking conclusive evidence of a benefit [9]. In all 24 patients of this analysis, an abdominal sub-hepatic drainage was implanted postoperatively, in accordance with the protocol of our center recommending the use of a drain in patients at risk of bleeding or of biliary leakage. Absorbed hemostatic material can visibly alter the color and consistency of the drained fluid, which can appear darker and corpusculated, leading to a wrong diagnosis of bile leakage or abscess. Also for this reason the use of ORC should be notified to clinicians treating the patient after cholecystectomy.

### Conclusions

In high-risk patients undergoing laparoscopic cholecystectomy with bleeding not adequately controlled by conventional techniques, ORC was easy to use and effective in ensuring adequate hemostasis. The role of topical hemostatic agents, including ORC, should be further investigated in prospective randomized studies with larger sample sizes in order to understand their potential and to optimize their use.

## Limitations

The retrospective nature and small sample size of this study limit generalization of our data. Nevertheless we believe the present analysis provides useful information regarding the use of ORC in a heterogeneous population typically encountered in clinical practice, and in laparoscopic cholecystectomy interventions at increased risk of bleeding complications.

## Abbreviations

CT: computed tomography
ORC: oxidized regenerated cellulose
